# The Patient Experience: Informing Practice through Identification of Meaningful Communication from the Patient’s Perspective

**DOI:** 10.3390/healthcare6010026

**Published:** 2018-03-20

**Authors:** Angela Grocott, Wilfred McSherry

**Affiliations:** 1 The University Hospitals of North Midlands NHS NHS Trust, Newcastle Rd, Stoke-on-Trent ST4 6QG, UK; W.McSherry@staffs.ac.uk; 2 Department of Nursing, School of Health and Social Care, Staffordshire University, Blackheath Lane, Stafford ST18 0YB, UK; 3 VID vitenskapelige høgskole, Haraldsplass Bergen, Ulriksdal 10, 5009 Bergen, Norway

**Keywords:** communication, patient experience, patient satisfaction, engagement, involvement

## Abstract

(1) Background: There is limited empirical knowledge concerning aspects of healthcare that contribute to a good patient experience from the patient’s perspective and how patient feedback informs service development. (2) Aim: To examine the issues that influence the effectiveness of communication on patient satisfaction, experience and engagement, in an acute National Health Service (NHS) setting, through identification of the patient’s requirements and expectations. (3) Method: Data was gathered from a large teaching hospital using a Friends and Family Test (FFT) and a communication specific survey. Both surveys captured patient narrative to identify predominant influences to explain the quantitative responses. (4) Results: The key priorities for patients are involvement in their care and receiving the right amount of information to support this. However, the delivery of compassionate care was identified as having the most influence on the likelihood of patients to recommend an acute NHS Trust. (5) Conclusion: The findings support a broader understanding of the constituents of an all-encompassing patient experience from the patient’s perspective. (6) Implications: healthcare organizations need to focus their resources on how to improve patient/provider communication to support patients to be true partners in their care.

## 1. Introduction

Patient participation in healthcare decision-making is part of a wider trend towards a more bottom-up approach to service planning and delivery with patient experience increasingly conceptualised as a fundamental measurement of healthcare quality [1], patient safety and clinical effectiveness [2]. This has resulted in the introduction of politically driven surveys to measure patient satisfaction and to evaluate the degree to which care is patient-centred [3]. 

The development of patient/healthcare partnership through reciprocal communication has the potential to strengthen therapeutic bonds [4]. However, to achieve this, it is imperative that patient expectations are considered with an awareness that patient and provider definitions of meaningful communication are likely to differ [5]. As there is a global scarcity of empirical studies that examine influential encounters in an acute healthcare setting [6], an aim of this study was to explore the attributes of meaningful communication in an acute healthcare setting from the patient’s perspective. The authors are aware that there is growing interest in the area of patient experience and this is reflected in the growing number of journals devoted specifically to this field, for example the *Journal of Patient Experience* (published by the Association for Patient Experience). 

Communication continues to figure consistently as a significant theme in both patient satisfaction and complaints about care delivery [7]. Gaining an understanding of preferred communication from the patient’s perspective has the potential to encompass the range of interactions that they prioritise and to take seriously the need for responsiveness to individuals [8]. Putting involvement at the forefront of policy and practice provides the opportunity not only to create an effective and sustainable health and care system, but also to contribute to a more equitable and healthier society [9]. The evidence shows that when patients feel they have a role to play in their care, decisions are better, health and health outcomes improve, and resources are targeted more efficiently [9]. 

Despite political drivers [10,11], to encourage patient feedback as a resource for the development of patient focused care delivery, National Health Service (NHS) patients continue to provide feedback indicating that they would like more information and greater opportunities to participate in decision making about their daily care and treatment [12,13]. There is limited evidence to indicate that this situation has improved over the last ten years demonstrating the need for further research [14]. 

As patient experience is multi-faceted, it is very difficult to develop an approach that suits all. However, learning what actually matters for patients during a time of acute illness provides a commonality, which has the potential to inform future practice [15] The majority of patients are naturally anxious about their illness and this is exacerbated when a trusting relationship has not been developed and the patient feels they are not provided with an opportunity for informed choice about treatment options [16]. 

It has long been argued that communication forms the foundation of all human interaction [17]. Gaining an understanding of preferred communication from the patient’s perspective has the potential to encompass the range of interactions that take priority from their perspective and to take seriously the need for responsiveness to individuals [8]. 

## 2. Literature Review

A review of existing literature was undertaken to examine current findings relating to the impact of communication on the patient experience from the patient’s perspective. The review revealed that there is a scarcity of available literature on this subject. From 2010 to 2012 the literature focused on the behaviours affecting a good patient experience. There was a shift in focus during 2014 as both [18,19] examined the characteristics of negative patient experiences. 

The key words used for the search were “Communication” and “Patient Experience” in the title or abstract. As the initial search provided a large number of inappropriate hits the words “Acute Care” and “Research” were added to provide articles which were more relevant to the aims of the evaluation. The following databases were searched indicating the number of hits on each site:

- Cinahl plus with full text (hits = 46)
- BMJ Journals online (hits = 47)
- Cochrane Library (hits = 69)
- Medline (hits = 3)
- Pro Quest Nursing and Allied Health Source (hits = 300)
- RCN Journal (hits = 35)
- Wiley online library (hits = 38)
- Google Scholar (hits = 96)

An inclusion and exclusion criteria was systematically applied resulting in 12 research studies for an empirical literature review. 

The search was restricted to papers published in English. Searches were also restricted to papers published from 2008 to date, to reflect relatively current experiences following the Lord Darzi report [20], which put patient experience on the Political agenda. The inclusion criteria specified that the article was a research paper which explored experience from the patient’s perspective and the patients were adults who were providing feedback on their experience in an acute hospital setting. Scanning of the reference lists identified a further 3 papers. 

As collecting feedback from NHS patients in England became routine during 2012, with the implementation of the Friends and Family Test (FFT) [21] there developed recognition that this feedback was often not shared or acted upon leading to research being conducted to identify how this could be encouraged [22]. Recognition of the benefits and importance of gaining patient feedback was driven by the introduction of the “Friends and Family Test” [21]. The Friends and Family Test (FFT) was piloted in many hospitals in April 2012 with at least ten percent of acute adult in-patients asked how likely they would be to recommend the hospital to their family and friends should they need similar treatment. The usefulness of the test, based on one used in the retail industry, has been challenged as inappropriate for use in a healthcare setting [23]. It has been suggested this quantitative data is not sophisticated enough to capture the specific personal issues that are important to the patients and it is argued data collected in this way is more meaningful to the provider than the patient [24,25]. 

The Picker Institute Europe [26] recently reviewed the FFT concluding that it is unsuitable to use as a performance indicator between Trusts with criticism of its methodology by researcher [27,28]. The combination of the varying collection methods used by individual Trusts (for example paper based, online, and text) and different patient demographic profiles of respondents has always had the potential to significantly skew the test’s results. It is argued therefore that these factors mean that true comparisons between Trusts are impossible [29]. 

There is recognition that patient narrative is a powerful tool to drive improvement when staff can see patient responses in their own words [30]. Many Trusts have introduced the opportunity for patients to explain why they have chosen their response to the FFT question and this was rolled out as a national requirement from April 2015. The addition of qualitative patient feedback has the potential to provide a richer conceptualisation of both negative and positive interactions and how these may be developed as experience perceptions are subjective and individual [14,15,16,17,18,19,20,21,22,23,24,25,26,27,28,29,30,31]. 

Relationship-centred care remains a key theme in contemporary healthcare with recognition that patients who are listened to feel more involved in their care and retain a sense of control [32]. Patients who understand the information provided by the doctor (the term doctor has been used as opposed to physician because this is the term used in the questions) feel a greater sense of control over the treatment decision reducing anxiety and increasing hopefulness making adherence to treatment more likely as they have been motivated by the promise of a positive outcome through the development of a trusting relationship [18]. 

Contemporary studies suggest that, when patient’s expectations and emotional needs are met, communication outcomes are enhanced [32]. However, it is difficult to determine the impact patient expectations have on the overall patient experience and the consequent feedback they provide. As patient experience is strongly linked to fulfilment of expectations, research is required to further examine how to measure expectations and their influences [33]. 

Limited resources feature highly in patient expectations with more patients reporting doctors and nurses often do not have sufficient time for communication affecting their ability to involve patients in their care and listen to their concerns [34]. As this is a subjective evaluation individual factors should be considered to accommodate individuality [35]. With recognition that people assign different weights to different experiences to arrive at an overall evaluation, customer satisfaction theoretical models have been successfully used to identify what matters most to patients through correlation of survey responses with the patient’s numerical rating of the quality of the care they received and their willingness to recommend [36]. 

It is recognised that a caring environment promotes patients’ awareness, resulting in reduced anxiety, improved self-esteem and feelings of being in control [6]. Caring behaviours have the most impact on patient satisfaction with the attributes of closeness, involvement, interaction and relationship being those most wanted by patients and interestingly these are the least observed [37]. 

A caring environment is the most influential when patients make judgements on their willingness to recommend the hospital [36]. The concept of caring staff incorporates a willingness to help and answer questions, responsiveness to requests, showing courteous behaviour, dignity and respect, providing clear explanations about medicines and how patients should care for themselves post discharge [36]. 

Authors have identified positive patient responses for older patients were around staff attitude and the most negative were food quality, noise and the inability to obtain help with poor communication cited as a contributing factor [34]. However, a high willingness to recommend score suggests that staff attitude may have the most influence on a good experience for older patients and the patients expect the nurses to have limited time to communicate as they are “too busy” reflecting the findings of [32] in relation to “busy” doctors.

## 3. Methods 

The literature demonstrates that although there are a variety of survey tools, these may be inadequate for measurement of the effects of communication on patient experience and the actions that influence this. However, some positive recommendations were identified and utilised in this investigation especially around data collection and analysis. For example, a communication-specific survey was adopted with recognition that a focus on an individual theme (communication) has the potential to refine the data collection [22]. Furthermore, measuring the quality of communication alongside willingness to recommend has the potential to provide generalisations which may support a strategy to recognise and manage individual expectations [36,37]. 

A major observation in the literature is that data collection tools are predominantly quantitative (using surveys) therefore this investigation encouraged patient narrative to demonstrate why they chose their specific responses to the questions asked. This method supplemented the wealth of data collection and is more likely to influence staff engagement [22]. 

It is argued that carrying out studies while the patient is still in hospital is more likely to provide feedback on how the patient is feeling at the time [33] and gain information on those aspects of patient experience the patients themselves see as important [14]. Despite this the literature review identified that the majority of studies examining patient experience of acute care are based on retrospective data. 

### 3.1. Aims

This investigation sought to go beyond what patients liked or did not like about their care in an attempt to identify how the experience made them feel and the contributing attributes of both a positive and negative experience. The aims were:

(1) Examine the issues that influence the effectiveness of communication on patient satisfaction, experience and engagement, in an acute National Health Service setting, through identification of the patient’s requirements and expectations.
(2) Explore the attributes of meaningful communication in an acute healthcare setting from the patient’s perspective.

### 3.2. Design

This investigation used a quantitative design comprising of two questionnaires each with free text boxes to encourage patient narrative. A key word analysis of patients’ free text responses was carried out to provide a meaningful picture of their perceptions, feelings and experiences in acute care [38]. Identifying emotions involved looking for words or phrases that describe the emotional impact of the patient experience in the data collected. The resulting commonalities and the frequency that they occur were then compared with the quantitative responses of the FFT and communication surveys. 

### 3.3. Sample and Settings

The data for this study was gathered from a large acute NHS Trust situated across two hospital sites Royal Stoke Hospital (Stoke-on-Trent) and County Hospital (Stafford). Between 5000 and 6000 adult inpatients are discharged each month all of whom should be provided with the opportunity to answer a short FFT satisfaction survey on the day of discharge. Encouragement to provide narrative is included within the survey by asking the responder, “What is the main reason for the answers you have chosen?” and “What could we do better?” The FFT survey responses were gathered via tablet or paper survey and the responses uploaded onto a secure data base (See Table 1). The responses from discharged patients throughout the month of September 2015 were analysed.

During the week commencing 15th September 2015, an additional survey containing 14 questions specifically about communication was hand delivered to all adult in-patients in the same acute Trust across both hospital sites. This survey also provided an opportunity for free text patient responses. The questions were adopted from the standard National Inpatient Survey questions designed by Picker Institute Europe, utilising a Likert scale for data analysis. The data generated using standardised National survey tools is generally of high quality, reliability and validity as these have been psychometrically tested [39].

### 3.4. Ethics

As this study was an evaluation of existing practices for capturing patient feedback, with no identifiable patient information, ethical approval was not required. This decision was confirmed by the local Research and Development Department at the University Hospitals of North Midlands NHS Trust who reviewed the proposal and associated documents. 

### 3.5. Data Analysis

The dependent variables for this study are: 

“How likely are you to recommend our ward to your friends and family if they needed similar care or treatment?”and, “Did you feel you were involved as much as you wanted to be in decisions about your care?”

The independent variable questions related to the characteristics most likely to influence the response were care and communication specific to enable the authors to identify the causal relationship between good communication and patient experience [40]. 

Optimum Contact Ltd., Meridian software was used to numerically weight the multiple-choice responses to each survey to identify the strength of agreement or disagreement with each question in line with the National Inpatient Survey methodology. Pearson’s statistical correlation [41] was used to calculate the importance of each question to the patient’s likelihood to recommend and feeling that they were involved in their care whilst in hospital (See Table 2 and Table 3).

The narrative feedback from both surveys was reviewed to gain an initial impression of the content. This was followed by a more in-depth review and analysis conducted with the aid of the Meridian software tool. The most commonly cited key words (See Table 4 and Table 5) were identified and used to classify and cluster the responses to summarise the data and identify categories. 

Concentration was focused on identification of the key words most commonly used by those patients who scored highest and those who scored lowest in each survey to examine the variation of behaviour and effect identified in the data. This approach allowed the authors to revisit the data to refine their understanding of the context in which these words were used. As the narrative responses were predominantly positive, the researcher scanned for those words that scored at least equal to the average FFT weighted response score and occurred more than 20 times (Table 5). Scanning the narrative feedback for those words that scored less than the average 94% weighted score resulted in identification of the key words used most by those patients who are least satisfied about their hospital stay. Due to the predominantly positive weighted narrative feedback there were only 2 words identified as appearing at least 20 times: “better” scoring 89.51% and “nurses” scoring 86.51%. The researcher therefore scanned for words written in a negative context on 5 or more occasions (Table 4). 

## 4. Results

### 4.1. The Friends and Family Test (FFT) Survey Results

A total of 1444 adult patients, age 18 years or over and spending at least 1 night in hospital, responded to the FFT Survey (out of 5354 discharges) providing a 26.9% response rate against an average national response rate of 25% for the same period [42]. For a breakdown of the individual responses, please see Table 1. Maternity patients were excluded as these patients complete a different FFT survey, which is relevant to their circumstance. 

The overall percentage satisfaction score demonstrated by patients likely or extremely likely to recommend the ward to their friends and family should they require similar care or treatment, was 98% exceeding the National score of 95% [42]. 

**Figure 1:**
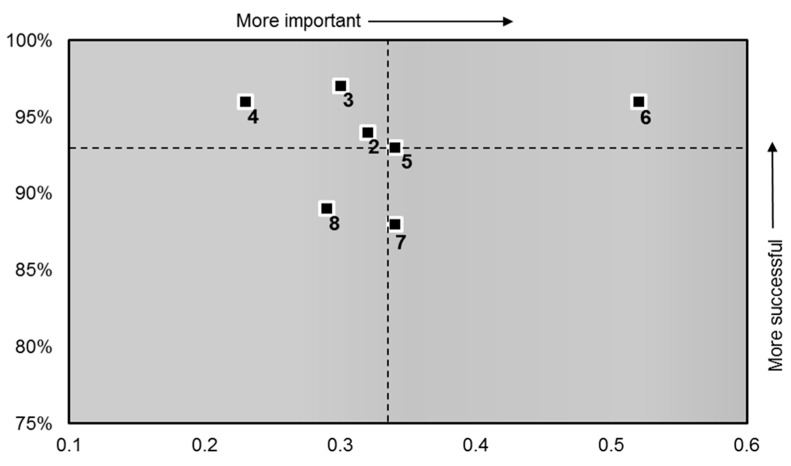
Scatter Diagram (FFT Survey).

The scatter diagram (Figure 1) presents the correlation between Question 1 “How likely are you to recommend our ward to your family and friends should they need similar care or treatment?” and the other 7 questions in the FFT survey. Each square on the chart represents an individual question from the survey. The position of these squares identifies which questions have the most influence on the patient’s likelihood to recommend the hospital. 

The results suggest that questions 5, 6 and 7 have the highest influence on a positive likely to recommend score. Questions in the upper left quadrant have an above average score and a low importance to the patient. Questions in the bottom left quadrant have a below average score and low importance. Therefore, these questions are less likely to influence the patient’s willingness to recommend the hospital. 

The question results are displayed in Table 2 show the co-ordinates used to plot the scatter diagram (Figure 1)

### 4.2. The Friends and Family Test (FFT) Narrative Analysis

Analysis of the narrative feedback resulted in the identification of the 5 key words that contributed to the overall FFT likely to recommend score (Table 2) and the 4 words that contributed towards the lowest scores (Table 5).

### 4.3. The Communication Survey Results

The Communication Survey generated a 39% response rate, with 1510 surveys distributed and 591 returned. 

Question 10 “Did you feel you were involved as much as you wanted to be in decisions about your care?” was used as the independent variable on which to measure the relationship between the other 13 questions and the patient’s perception of feeling involved in their care. 

**Figure 2:**
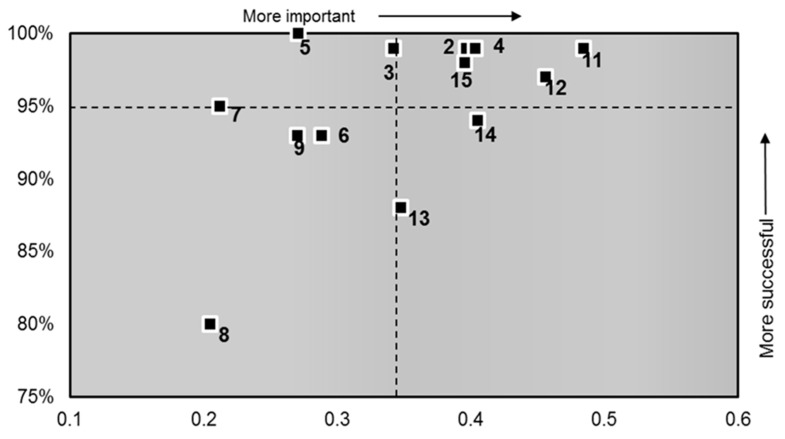
Scatter Diagram (patient-to-staff communication).

The results suggest that questions 2, 4, 11, 12, 13, 14 and 15 have the highest patient priority when measured against their perceived involvement in their care (Figure 2). 

Table 4 shows the patient to staff communication results in order of importance to the patient and the coordinates used to plot the diagram.

### 4.4. The Communication Survey Narrative Analysis

Analysis of the narrative feedback—provided in the Communication survey—identified that the word “communication” was used 27 times. Twelve of these were in a negative context with patients either stating they would have liked more communication with the health professional or better quality communication. 

Analysis of both surveys:

An in-depth analysis of the negative comments/suggestions for improvement in both surveys resulted in the emergence of 3 key themes:

- 21 patients suggested that more staff were needed on the wards as they felt that the staff caring for them were too busy.
- 42 patients felt there was too little or inconsistent communication about their condition and/or hospital stay
- 22 patients described frustrations with delays resulting in a longer hospital stay after they had been told they could go home.

## 5. Discussion

The words “compassion” and “compassionate” were used positively in 15 responses and the words “care” or “caring” used positively 133 times in the patient narrative of both surveys. This study has identified positive attributes of staff described as “professional,” “kind,” “friendly” and “helpful” alongside “caring.” It may therefore be presumed that these are the key characteristics which lead to patients feeling they have been treated with compassion. 

It has been argued that patient experience measures the structures and processes of care based on expectations [43]. Consistent with the findings of [34] the written narrative of respondents identified an impression of limited resources in the NHS. However, this does not affect their overall likely to recommend score. The FFT scatter diagram (Figure 1) suggests patients may have a preconceived expectation that, as they are likely to be cared for in a shared room, their privacy and dignity is at risk of compromise. This is demonstrated by the fact that although question 3—“Do you feel your privacy and dignity was respected?”—scores relatively high, it is situated 5th in the importance ranking and in the left quadrant, and is therefore less influential on the FFT score than may be expected. 

Questions 5 and 7 are situated in the right-hand quadrant (Figure 1), indicating that patients do want more involvement in decisions about their care, treatment and discharge. Despite their ranking as 2nd and 3rd importance and the position on the chart indicating these two subjects should be the main focus for improvement, this has not influenced the overall “likely to recommend” score. Interestingly, this does influence the National Inpatient Survey results on which the communication survey results are based [13].

Although a specific question is not asked about the numbers of staff on the ward in the FFT survey a perception of too few staff emerges as a theme in the patient narrative. The communication scatter diagram (Figure 2) identifies question 8, “In your opinion were there enough nurses on duty to care for you in hospital?” as the least important influence on the patient’s overall impression of receiving enough information about their care and treatment and being involved as much as they wanted to be in decisions about their care and treatment. This supports the findings of References [32,34]—that patients expect clinicians not to have the time to listen and involve patients in their care, perpetuating their reluctance to become actively engaged.

Rapport-building can be difficult as time constraints for busy clinicians often dictate a task focused approach with concentration on diagnosis and treatment to the detriment of ensuring individual patient concerns are addressed [44]. The high “likely to recommend” score suggests patients interpret this as expected behaviour. However, an environment where patients and relatives perceive staff are not readily available to respond to questions or requests leads to a loss of opportunity for partnership working by presenting a barrier to initiating communication [45]. The long-held belief that time is essential for meaningful conversation is challenged with the findings of this study that patients put more importance on a caring manner and a willingness to communicate demonstrated by kind, friendly staff. 

Although 73% of patients and carers responded that they always received answers to important questions from the doctor in a way they could understand the 26% “sometimes” responses suggest that a number of patients are seeking more or clearer information. Doctors talking about the patient as if they were not there is a missed opportunity for information sharing with only 66% of patients reporting that they were always included in conversation with their doctor and 77% receiving enough information about their condition or treatment. Although there are some studies that suggest the time spent during communication between clinician and patient has a direct influence on the quality of the information receive [46] there are studies which argue that the content of the communication and the ability to listen outweighs any benefit which may be gained through time spent [19].

Of the FFT respondents, 83% were involved as much as they wanted to be in decisions about their care and this reduced further to 77% for involvement in discharge decisions. Although this did not affect the likely to recommend score it highlights a need for improved engagement and communication with patients. 

The scatter diagrams (s 1 and 2) demonstrate key priorities for patients who expect and want to be involved in their care and receive the right amount of information to enable this. They want to receive answers to their questions in a way they can understand, have any tests and/or procedures explained and have confidence and trust in the doctors treating them. These results suggest patients want to be provided with the opportunity to be actively engaged contrary to the belief of many clinicians [47]. 

With a political drive to encourage and support the public to be more actively engaged in decisions about their own health [10], it is important to ensure their expectations are realistic and opportunities for communication are sought without creating barriers to potential patient led improvements [48]. An ability to communicate in a manner, which identifies the situation from the patient’s perspective, is arguably the pivotal skill required to enable this [49] with recognition this is particularly difficult when personal characteristics or beliefs differ. 

This study suggests that patients want to be afforded the opportunity to be actively engaged however their expectations are often linked to past experience of self or others. They are understandably often anxious, finding themselves in strange surroundings reliant on unfamiliar staff and are reluctant to ask questions for fear of being considered a difficult patient [50], with an expectation that the healthcare professionals will be too busy to answer their questions. 

Encouraging patients to share decision-making, alongside the professionals caring for them, requires interventions aimed at changing long established behaviours and perceptions of both staff and patients [47]. Contemporary healthcare is evolving with a change in attitudes from a paternalistic approach which some patients and staff find difficult [51]. Health Professionals who provide tools to support understanding and encourage patients to ask questions are more likely to tailor information sharing to individual needs [10]. Patients who have the opportunity to communicate are more likely to have realistic expectations around their care and prognosis. However, to reduce barriers, this must be at the patient’s own level of understanding and at the most appropriate time [52] with consideration for the effect of acute illness on engagement. 

## 6. Conclusions

The findings indicate that it is the responsibility of all healthcare providers to improve their communication skills, demonstrating a willingness to communicate by proactively encouraging patients to ask questions. Providers may also need to promote extended visiting hours to support more opportunity for communication. Finally, there is a need to ensure patient information leaflets are written to comply with national guidance to promote understanding [52]. As long as the patient remains dissatisfied with the communication they have received there will remain opportunity for improvement through a patient-centred approach. This will only be achieved when the caring encounter is experienced as a meaningful encounter. Patients who understand the information given to them and are given a sense of control in the decision-making process are likely to be less anxious and comply with treatment recommendations [18] with potential to reduce the length of stay and risk of readmission. Despite the limitations of this study, it has provided a foundation for future research in this field through the identification of influencing factors that contribute to overall patient satisfaction and the difficulties surrounding patient understanding and engagement. 

## 7. Implications for Practice

The results from this evaluation can be used to develop a culture that encourages patients to ask questions by:

- Developing an inpatient leaflet which explains the concept of shared decision making and why it is important. Provides an explanation that their knowledge about their health and lifestyle is as important as the clinician’s expertise with each complimenting each other.
- Creating a communication drive to encourage patients to ask questions providing suggested questions as examples on electronic posters, notice boards and hospital websites
- Introducing a paper at the bedside for question prompt lists to enable questions to be written down as the patient and/or relative thinks of them in preparation of ward rounds
- Exploring the use of the internet as a patient information tool to generate questions.
- Supporting clinicians to improve their communication skills—Develop a training programme for introduction of “teach back” methodology
- Promoting extended visiting hours to support more opportunity for communication.
- Ensure patient information leaflets are written to the recommended reading age to facilitate understanding by the majority of the population.

## 8. Recommendations for Future Evaluations/Research

A significant limitation of this research is its cross-sectional nature, meaning that the patient’s experiences were captured at a single time point and with a specific cohort or group of patients. This type of evaluation may be better conducted more longitudinally. Similarly, patients may report more positive experiences when completing surveys while in hospital, just prior to discharge.

- Conduct the evaluation for planned and emergency admissions separately to identify if there are any variances in results
- Repeat at ward level to identify examples of good practice for dissemination across the organisation
- Repeat with inclusion of demographic detail to identify if there are any variances in results.
- Repeat for individual demographic patient groups to identify specific communication strategies and needs.

## Figures and Tables

**Table 1:** Meridian Desktop, Friends and Family Test (FFT) Results.

1	How likely are you to recommend our ward to your family and friends if they need similar care or treatment?	Extremely likely	Likely	Neither likely nor unlikely	Extremely unlikely	Don’t know	Comments
		1151	261	4	3	2	0
2	Do you feel your pain was kept under control?	Yes always	Yes sometimes	Not at all	Never had pain	Comments	
		1166	150	9	119	0	
3	Do you feel your privacy and dignity were respected?	All of the time	Most of the time	Some of the time	None of the time	Comments	
		1328	92	22	2	0	
4	Did you get enough help from staff to eat your meals?	Yes always	Yes sometimes	Not at all	Did not need help	Comments	
		761	45	7	630	0	
5	Were you involved as much as you wanted to be in decisions about your care and treatment?	Yes	Most of the time	Sometimes	Never	Not applicable	Comments
		1195	177	57	9	5	0
6	Did you feel that you were treated with compassion?	All of the time	Most of the time	Some of the time	None of the time	Not applicable	Comments
		1285	122	23	1	13	0
7	Did you feel you were involved in decisions about you discharge from hospital?	Yes definitely	Yes, to some extent	No	I did not need to be involved	Not applicable	
		1065	248	41	26	64	0
8	Were you given enough notice about when you were going to be discharged?	Yes definitely	Yes, to some extent	No	Not applicable		
		1086	241	34	80	0	
9	What was the best thing about your experience today?	Comments					
		584					
10	What one thing could we have done better?	Comments					
		169					
The following link provides all the information and guidance about the Friends and Family Test https://www.england.nhs.uk/fft/							

**Table 2:** Friends and Family Test questions most likely to influence a likely to recommend score.

Question Number	Question in Order of Importance	Score	Importance
6	Did you feel you were treated with compassion?	96.08	0.52
7	Did you feel you were involved in decisions about your discharge from hospital?	87.82	0.34
5	Were you involved as much as you wanted to be in decisions about your care and treatment?	92.69	0.34
2	Do you feel your pain was kept under control?	93.65	0.32
3	Do you feel your privacy and dignity were respected?	96.75	0.30
8	Were you given enough notice about when you were going to be discharged?	88.73	0.29
4	Did you get enough help from staff to eat your meals?	96.36	0.23

**Table 3:** Communication Survey questions most likely to influence patients feeling involved in decisions about their care.

Question Number	Question in Order of Importance (Patient to Staff)	Score	Importance (r)
11	How much information about your condition or treatment has been given to you?	99%	0.48
12	Has a member of staff answered your questions about the operation or procedure? (if applicable)	97%	0.46
14	Afterwards, did a member of staff explain the operation or procedure? (if applicable)	94%	0.41
4	Did you have confidence and trust in the doctors treating you?	99%	0.40
2	When you have important questions to ask a doctor do you get answers that you can understand?	99%	0.40
15	Do you feel you were given enough privacy when discussing your condition or treatment?	98%	0.40
13	Have you been told how you will feel after you had the operation or procedure? (if applicable)	88%	0.35
3	When you have important questions to ask a nurse do you get answers that you can understand?	99%	0.34
6	Do doctors talk in front of you as if you weren’t there?	93%	0.29
5	Did you have confidence and trust in the nurses treating you?	100%	0.27
9	Does one member of staff say one thing and another say something different regarding your care?	93%	0.27
7	Do nurses talk in front of you as if you weren’t there?	95%	0.21
8	In your opinion, are there enough nurses on duty to care for you in hospital?	80%	0.20

**Table 4:** Friends & Family Test Questionnaire, Words contributing towards the lowest scores.

Word	Word Count	Average Score	Negative Context
Discharge	17	82.86%	15
Waiting	7	82.36%	7
Communication	11	81.74%	8
Night	15	70.34%	10

**Table 5:** Friends & Family Test Questionnaire, Top scoring staff attributes.

Attribute	Word Count	Average Score
Professional	23	98.40%
Kind	28	95.99%
Friendly	61	95.41%
Caring	65	95.22%
Helpful	61	94.58%
